# Effect of Smell and Taste of Milk on Feeding Parameters in Preterm Neonates: An Updated Meta-Analysis

**DOI:** 10.7759/cureus.76110

**Published:** 2024-12-21

**Authors:** Sarah Alenezi, Manal Aldaihani, Sabah Alqabandi, Ahmad A Alkandari, Bader A Almukaimi, Latifah Almutairi, Mohamed Abualqassim, Ziad A Kanaan, Manaal H Ameen, Yara H Farahat, Ahmed Abu-Zaid

**Affiliations:** 1 Department of Medicine and Surgery, Kuwait Institute for Medical Specializations, Kuwait City, KWT; 2 College of Medicine and Medical Sciences, Arabian Gulf Univesity, Manama, BHR; 3 College of Medicine, Alfaisal University, Riyadh, SAU; 4 College of Medicine, Fatima Jinnah Medical University, Lahore, PAK

**Keywords:** gestational age, preterm infant, smell, sucking feed, taste

## Abstract

Smell and taste sensations have been linked to positive outcomes in the feeding of premature infants, though the impact on the time required to transition to oral feeding remains unclear. This study aimed to evaluate the beneficial effects of smell and taste interventions on clinical outcomes in preterm infants. We conducted a search in PubMed, Scopus, Web of Science, and the Cochrane Central Register of Controlled Trials from inception through September 2024 for randomized controlled trials (RCTs) examining the effects of smell and taste on clinical outcomes in preterm infants with a gestational age of less than 34 weeks. The quality of the included studies was evaluated using the updated Cochrane's Risk of Bias tool (version 2). The primary outcome was the time required to achieve oral feeds. Secondary outcomes included the time to reach full enteral feeds, length of hospital stay, postmenstrual age, the need for parenteral nutrition, and the incidence of nosocomial infections. The outcomes were summarized as mean difference (MD) or odds ratio (OR) with 95% confidence intervals (CI) using a random-effects model. A total of 12 RCTs involving 1,638 preterm infants were included in the analysis. The results showed that smell and taste interventions significantly reduced the time needed to reach oral feeds (MD = -1.37 days, 95% CI [-2.26, -0.48], p < 0.001; I² = 42.15%) compared to no intervention. These findings were consistent across subgroup analyses based on birth weight at admission, type of exposure, and sample size. However, no significant differences were found for the other secondary outcomes. In conclusion, smell and taste interventions significantly reduced the time to reach oral feeds, with similar outcomes for other clinical measures compared to no intervention. These findings suggest that smell and taste interventions could be used in the care of preterm infants, with the need for large-volume RCTs and long-term assessments being warranted.

## Introduction and background

Preterm infants are those born alive before 37 weeks of gestation. In 2020, an estimated 13.4 million newborns were born preterm, which is roughly 1 in 10 births. In 2019, around 900,000 children died from complications related to preterm birth [1]. Any of these deaths are preventable with appropriate medical care, particularly in low- and middle-income countries, where survival rates for premature babies vary significantly. For instance, more than 90% of extremely preterm infants (born at less than 28 weeks) in low-income countries do not survive their first few days, while in high-income countries, fewer than 10% of these infants die [1,2]. Additionally, even those who do survive may face risks of developmental delay [3], cardiometabolic diseases, and other disorders [4,5]. Proper nutrition is crucial for the growth and development of preterm infants, as improved weight gain and head growth are linked to better health outcomes and enhanced long-term neural development [6].

Preterm infants have an immature gastrointestinal system, and they show uncoordinated neonatal reflexes, such as sucking and swallowing; thus, they receive their eternal feeds through a nasogastric tube after failure of initial supply with parenteral nutrition. Prematurity in the gastrointestinal canal, sucking, and swallowing are the main causes of the higher need of using tube feeding; in addition, non-invasive interventions in the NICU, such as nasal continuous positive airway pressure and high-flow nasal cannula, could mandate the need for tube feeding as well [7]. Moreover, early initiation of enteral feeds has a great impact on preterm infants' health by promoting micronutrient delivery, intestinal development, and full maturation, reducing inflammation, and stimulating the maturation of intestinal microbes [8].

Smell and taste play a crucial role in digestion by triggering various physiological responses that prepare the body for food intake [9]. These sensations are mainly processed in the olfactory and gustatory cortices and are then integrated with higher brain functions [9]. In the context of preterm infants, smell and taste are often overlooked during tube feeding. However, these senses are known to enhance gut motility and stimulate the secretion of digestive enzymes, and may aid in promoting the release of digestive hormones. This systematic review and meta-analysis aimed to evaluate whether exposure to the smell or taste of breast milk or formula - administered through tube feeds - can facilitate the time needed to transition to oral feeds.

## Review

Methods

This systematic review and meta-analysis was performed according to the Preferred Reporting Items for Systematic Review and Meta-Analysis (PRISMA) [10] and the guidelines of Cochrane Handbook of Systematic Reviews and Meta-Analyses [11]. The research protocol was registered in the International Prospective Register of Systematic Reviews (PROSPERO, identifier: CRD42024603689).

We searched four databases (PubMed, Scopus, Web of Science, and Cochrane Central) for randomized controlled trials (RCTs) from inception till September 2024 using the following search strings: ((preterm infants) OR (pre-term)) AND ((smell sense) OR (taste sense)) AND ((infant formula) OR (baby formula) OR (breast milk)). The duplicates were removed by EndNote [12], and remaining citations were uploaded to Rayyan [13]. Furthermore, a manual citation analysis from published reports and past meta-analyses was performed to include all relevant studies.

RCTs involving preterm infants aged 24-34 weeks of gestation were considered eligible if the intervention focused on smell or taste, compared to a no-smell or no-taste control, and studied outcomes of interest using an intention-to-treat analysis. Moreover, we excluded observational studies, single-arm studies, or studies with unpublished data. Additionally, we excluded full-term neonates, neonates with chromosomal abnormalities, infants with neonatal seizures, neonates with need for mechanical ventilation, or neonates who had cranial bleeding or hyperbilirubinemia. Next, we performed a two-step approach during the literature search using Rayyan software [13]. The first step was title and abstract screening for all citations, and, subsequently, full-text screening was performed to include studies matching our inclusion criteria. Two independent authors executed the screening phases, and any disagreements were resolved via a consensus with a third author.

The primary outcome of interest was time to reach oral feeds defined as the time needed to remove the feeding tube. Other secondary outcomes were time to reach full enteral feeds defined as 150 mL/kg/day, duration of parenteral nutrition defined as the time needed to remove the intravenous (IV) nutrition line, length of hospital stay, first discharge post-menstrual age, discharge weight, and the incidence of necrotizing enterocolitis by positive culture test. We used an offline Excel sheet (Microsoft Corp., Redmond, WA) for data extraction. The extracted data were as follows: baseline characteristics of the included patients, first author’s last name, year of publication, study design, country and duration of study, sample size, study group, main inclusion criteria, and primary and secondary outcomes.

Two authors independently assessed the quality of the included RCTs using the Cochrane Risk of Bias 2 (ROB-2) tool for RCTs [14], which comprises five domains: randomization process, deviation from intended intervention, assessment of outcome measurement, missing outcome data, and selection of reported results. The decisions were categorized as “high risk of bias,” “some concerns,” and “low risk of bias.” Any conflicts were resolved via a discussion with a third author. Funnel plots were used to assess for publication bias, and trim and fill methodology was used in case of publication bias.

The continuous data expressed as mean and standard deviation (SD) were pooled as mean difference (MD) with its 95% confidence interval (CI) using the DerSimonian Laird random effect model. Moreover, the pooled dichotomous data expressed as event and total number of patients were analyzed using the odds ratio (OR) with its 95% CI using the random-effects model. Heterogeneity among studies was assessed using the I^2^ statistic and p-value, of which an I^2^ > 50% and a p-value of <0.05 indicated significant heterogeneity. We performed a Galbraith plot to visualize the heterogeneous studies using the overall standard error. Additionally, we performed subgroup analyses to the primary outcome of interest based on the type of exposure (only smell of milk or smell and taste of milk), the mean birth weight at admission (<1,500 g or >1,500 g), and sample size of each study (<100 participants or >100 participants). A sensitivity analysis model named “leave-one-out” was performed to assess the robustness of the evidence, of which multiple scenarios were performed by excluding one study at a time, to ensure that the overall effect estimate was not heavily related to a single study. All statistical analyses were performed using STATA MP18 for Mac (StataCorp LLC, College Station, TX).

Results

**Figure 1:**
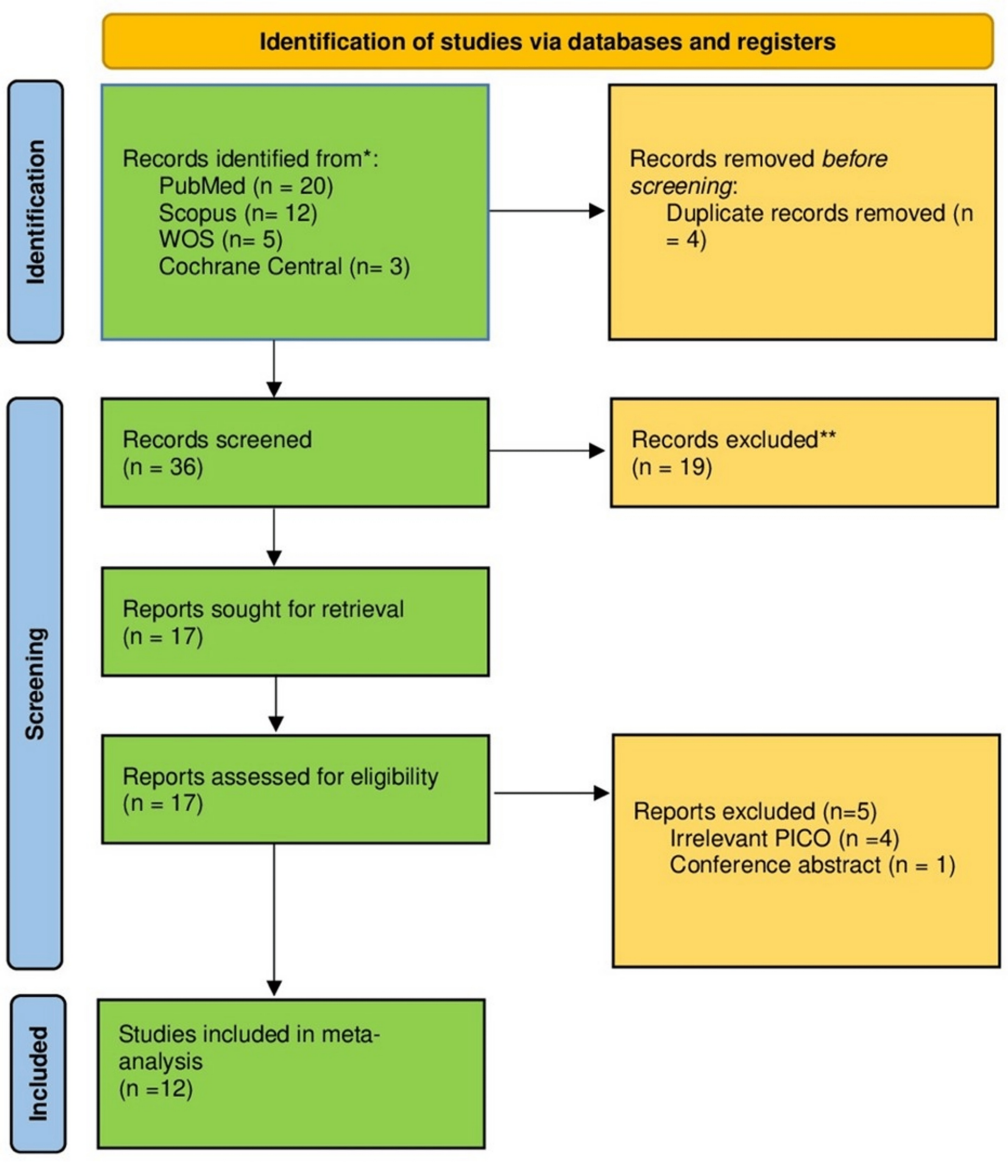
The PRISMA flow diagram for literature search and study selection. *Databases were screened from inception till September 2024. **Citations were excluded based on screening titles and abstracts. PRISMA, Preferred Reporting Items for Systematic reviews and Meta-Analyses

A total of 40 citations were retrieved from electronic search. After screening, 17 studies were assessed for eligibility, of which 12 RCTs that met our inclusion criteria were included in the meta-analysis [15-26]. The study selection process is illustrated in Figure 1.

We included 12 RCTs involving 1,638 preterm infants. The mean gestational age of the included infants ranged from 26.9 to 34.1 weeks. The geographical distribution, a summary of the included studies, and the baseline characteristics of the patients are presented in Tables 1, 2.

**Table 1:** Summary of the included studies. PMA, postmenstrual age; RCT, randomized controlled trial

Author, year	Country	Study design	Sample size (intervention/control)	Intervention	Outcomes
Yildiz et al., 2011 [21]	Turkey	RCT, two parallel groups	40/40	Only smell of milk	Time to full enteral feeds, length of stay in hospital, discharge weight
Iranmanesh et al., 2014 [17]	Iran	RCT, two parallel groups	46/46	Only smell of milk	Transition time from gavage to oral feeding (days), duration of hospital stay
Beker et al., 2017 [15]	Australia	RCT, two parallel groups	28/23	Smell and taste	Time to full enteral feeds, duration of parenteral nutrition, necrotizing enterocolitis, spontaneous intestinal perforation, discharge weight, PMA at discharge
Khodagholi et al., 2018 [19]	Iran	RCT, two parallel groups	16/16	Only smell of milk	Length of stay in hospital, discharge weight, PMA at discharge
Davidson et al., 2019 [22]	USA	RCT, two parallel groups	17/16	Only smell of milk	Time to full enteral feeds, PMA at discharge
Küçük Alemdar and İnal, 2020 [16]	Turkey	RCT, two parallel groups	30/30	Smell and taste	Time to transition to oral feeding (days), weight, height, head circumference, physiological parameters (oxygen saturation, peak heart rate, respiratory rate)
Beker et al., 2021 [20]	Australia	RCT, two parallel groups	196/199	Smell and taste	Time to full enteral feeds, duration of parenteral nutrition, necrotizing enterocolitis, spontaneous intestinal perforation, discharge weight, PMA at discharge
Alexander et al., 2024 [18]	New Zealand	RCT, two parallel groups	260/272	Smell and taste	Transition to full oral feeding, height/weight at discharge, hospitalization time
Le et al., 2018 [26]	China	RCT, two parallel groups	27/29	Smell and taste	Time to full enteral feeds, duration of parenteral nutrition, duration of gastric tube placement, length of stay in hospital, necrotizing enterocolitis, spontaneous intestinal perforation
Xu et al., 2022 [23]	China	RCT, two parallel groups	57/57	Smell and taste	Time to full enteral feeds, duration of parenteral nutrition, duration of gastric tube placement, length of stay in hospital, discharge weight
Lee, 2019 [25]	Korea	RCT, two parallel groups	12/16	Only smell of milk	Transition to full oral feeding, height/weight at discharge
Le et al., 2021 [24]	China	RCT, two parallel groups	89/76	Only smell of milk	Transition to full oral feeding parenteral nutrition time, hospitalization time

**Table 2:** Summary of the baseline characteristics of the included studies. APGAR, Appearance, Pulse, Grimace, Activity, and Respiration

Author, year	Male, n (intervention/control)	Birth weight (g), mean (intervention/control)	Gestational age, weeks (intervention/control)		APGAR score at 1 minute (intervention/control)	APGAR score at 5 minutes (intervention/control)
Yildiz et al., 2011 [21]	18/24	1,466.38/1,606.38	31.05/31.27		6.58/6.25	8.88/8.50
Iranmanesh et al., 2014 [17]	22/24	1,601.96/1,469.02	30.7391/30.3913		7.6087/7.5652	1.1129/8.7391
Beker et al., 2017 [15]	16/9	937/942	26.79/27.2		Not available	9/7
Khodagholi et al., 2018 [19]	7/7	1,320.9/1,311.2	29.714/30.142		Not available	Not available
Davidson et al., 2019 [22]	3/4	Not available	30.86/31		Not available	Not available
Küçük Alemdar and İnal, 2020 [16]	15/15	1,430.7/1503.8	30.26/30.25		6.40/6.25	7.53/7.37
Beker et al., 2021 [20]	100/104	950/929	27.5/27.6		Not available	8/8
Alexander et al., 2024 [18]	145/146	2,146.5/2098.1	33.8/33.8		Not available	Not available
Le et al., 2018 [26]	24/28	1,689.89/1689.89	Not available		Not available	Not available
Xu et al., 2022 [23]	63/63	1,670/1,670	Not available		Not available	Not available
Lee, 2019 [25]	10/11	1,456.17/1,433.13	30.2/29.93		5.83/6.13	7.50/8.13
Le et al., 2021 [24]	Not available	1,476.2/1,455.1	Not available		Not available	Not available

**Figure 2:**
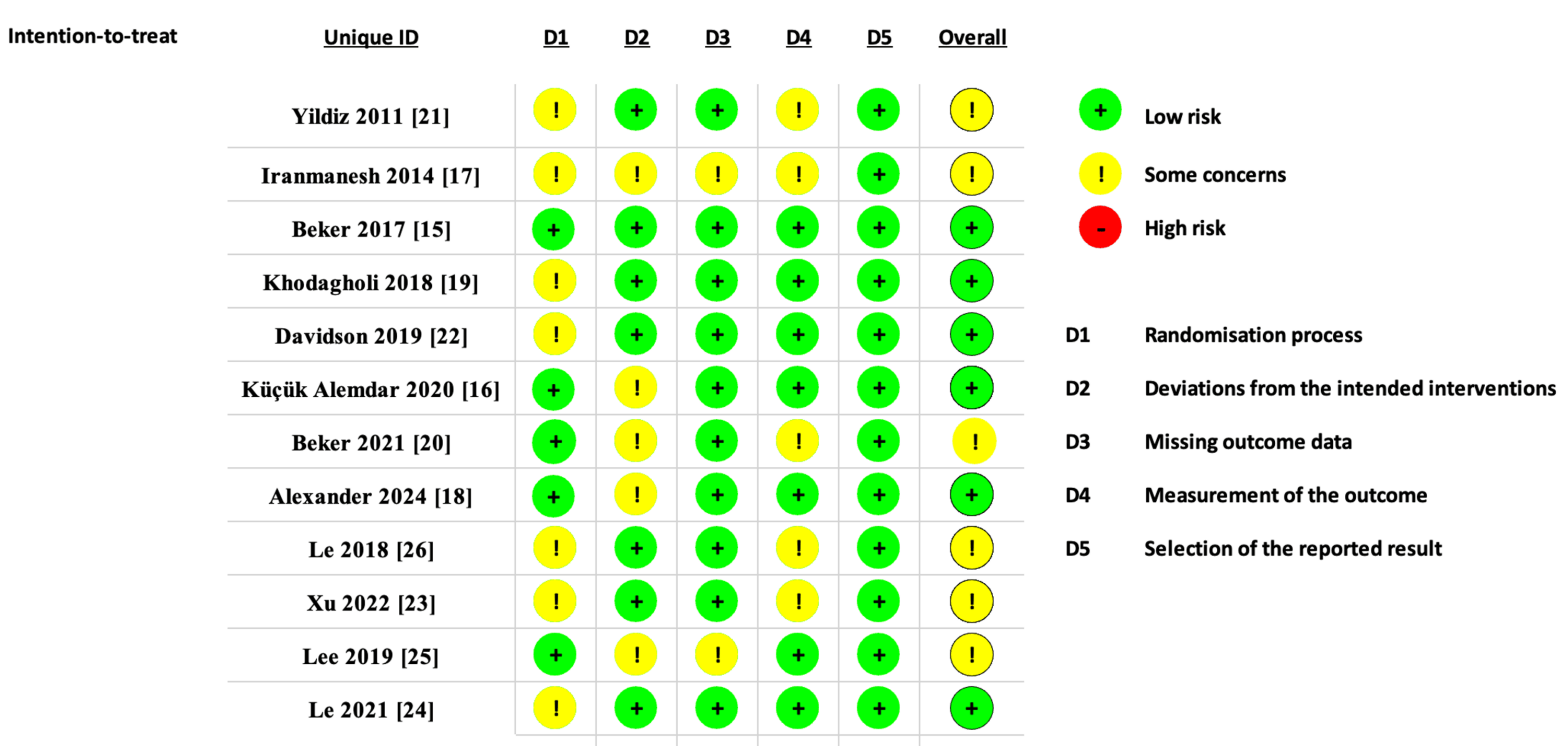
Summary of the risk of bias of the included studies.

Six RCTs were rated as low risk of bias, while six were rated as having some concerns regarding bias. Seven RCTs raised concerns about randomization due to insufficient information, while five studies raised concerns about deviations from the intended intervention. A detailed ROB-2 analysis is provided in Figure 2.

**Figure 3:**
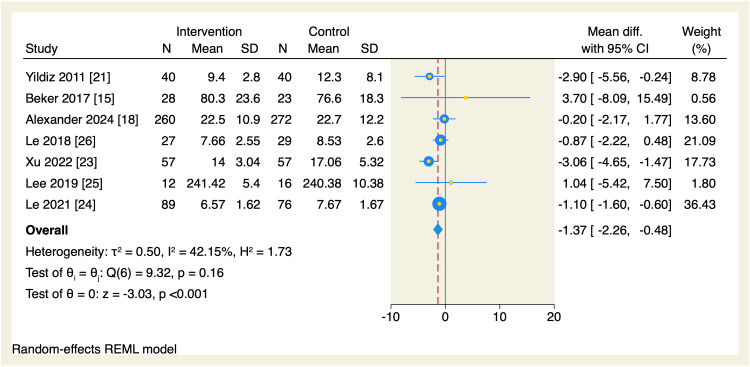
Forest plot of the time needed to reach full sucking feeds. CI, confidence interval; Diff., difference; N, sample size; REML, restricted maximum likelihood; SD, standard deviation

**Figure 4:**
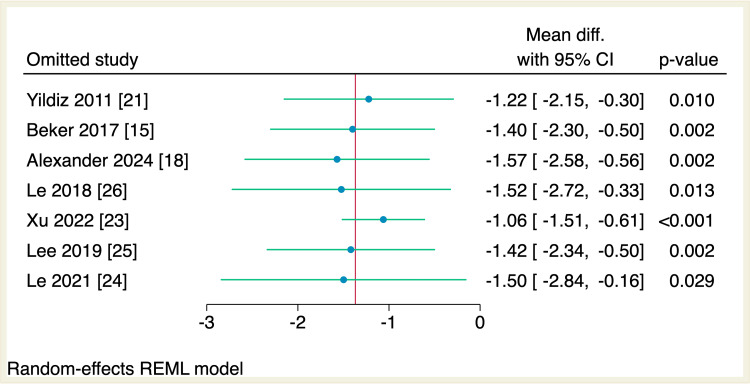
Leave-one-out sensitivity analysis plot of the time needed to reach full sucking feeds with 95% CI. CI, confidence interval; Diff., difference

Seven RCTs assessed the mean difference in the time needed to reach oral feeds among the two studied groups. The total amount of time needed to reach oral was significantly reduced in the intervention group compared to the control group (MD = -1.37 days, 95% CI [-2.26, -0.48], p < 0.001; I^2^ = 42.15), as shown in Figure 3. Leave-one-out sensitivity analysis was performed, which showed that no single study had a disproportional effect on the overall effect estimate, as shown in Figure 4.

**Figure 5:**
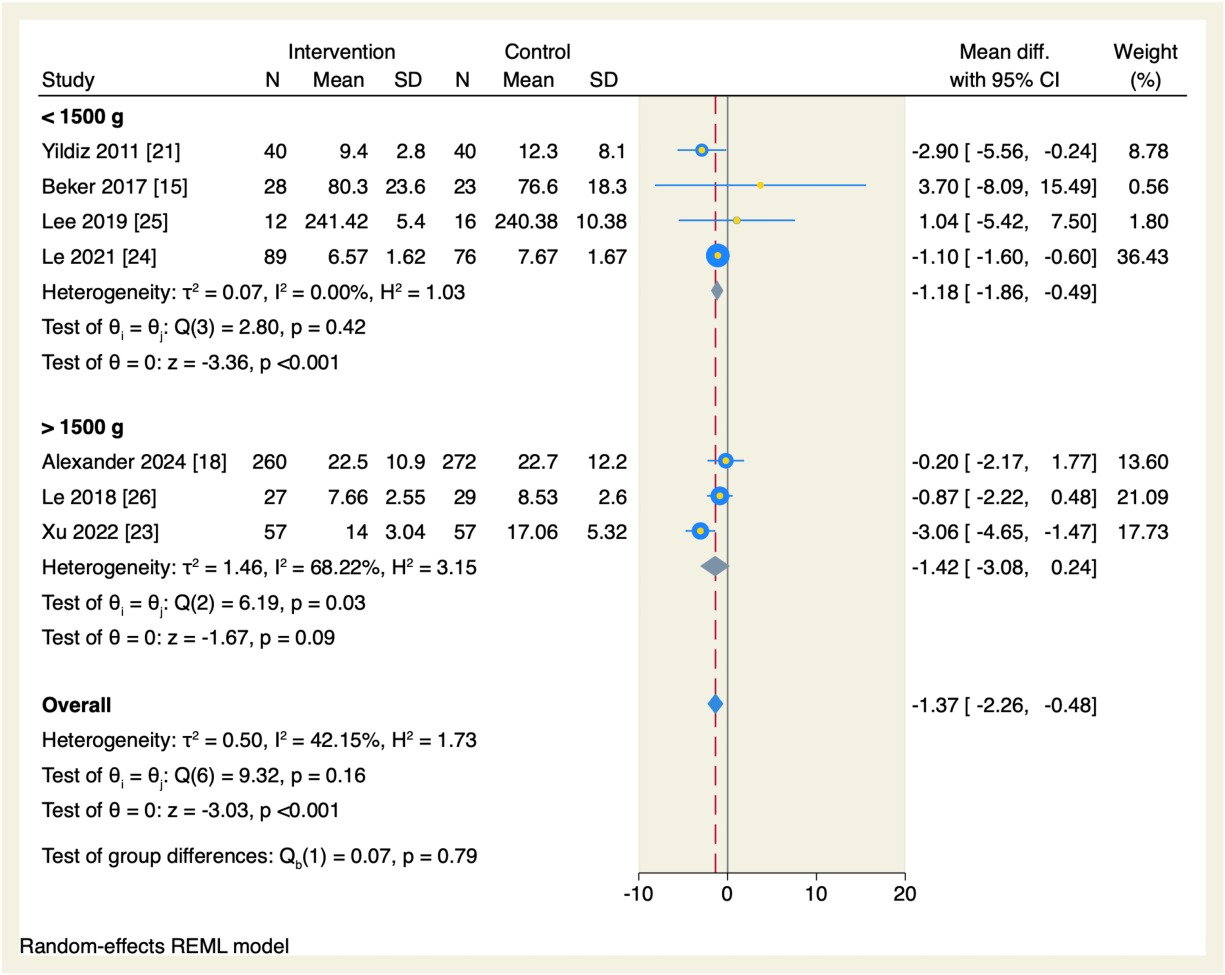
Forest plot of the time needed to reach full sucking feeds sub-grouped by the weight at admission. CI, confidence interval; Diff., difference; N, sample size; REML, restricted maximum likelihood; SD, standard deviation

**Figure 6:**
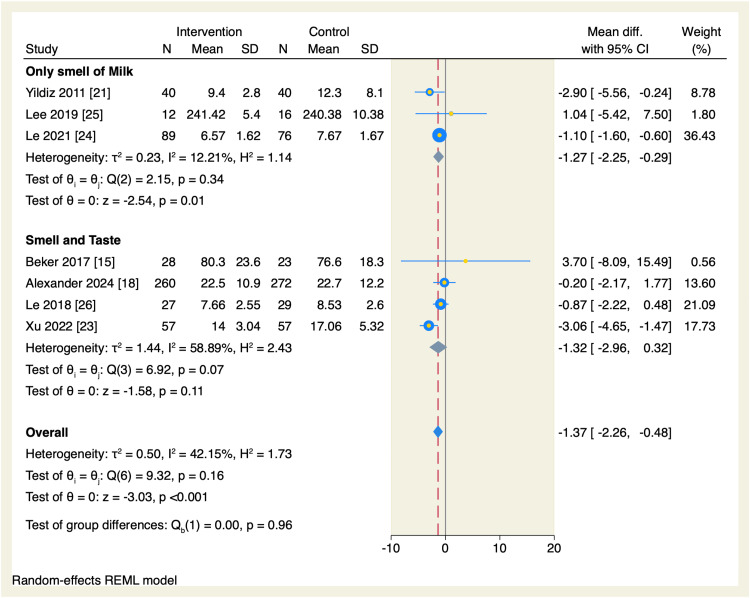
Forest plot of the time needed to reach full sucking feeds sub-grouped by the type of exposure. CI, confidence interval; Diff., difference; N, sample size; REML, restricted maximum likelihood; SD, standard deviation

We performed a subgroup analysis based on the weight at admission. The total amount of time needed to reach oral feeds was significantly reduced in the intervention group compared to the control arm in the subgroup of neonates <1500g (MD = -1.18, 95% CI: -1.86 to -0.49, p < 0.001; I^2^ = 0.00%), without significant difference in the neonates >1,500 g, as shown in Figure 5. Another subgroup analysis based on the type of exposure was performed, which showed that the subgroup of only the smell of milk favored the intervention group compared to the control group to decrease the amount of time needed to reach oral feeds (MD = -1.27 days, 95% CI [-2.25, -0.29], p = 0.01; I^2^ = 12.21%), without significant difference in the subgroup of smell and taste exposure, as shown in Figure 6.

**Figure 7:**
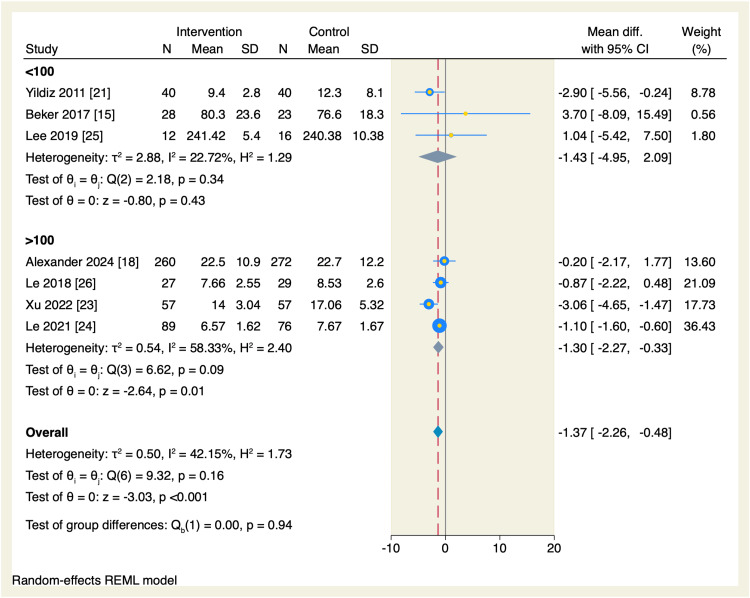
Forest plot of the time needed to reach full sucking feeds sub-grouped by the sample size of each study. CI, confidence interval; Diff., difference; N, sample size; REML, restricted maximum likelihood; SD, standard deviation

Additionally, we performed a subgroup analysis based on the sample size of each study. In the studies with >100 participants, the amount of time needed to reach oral feeds was significantly reduced in the intervention group compared to the control group (MD = -1.3 days, 95% CI [-2.27, -0.33], p = 0.01; I^2^ = 58.33%), as shown in Figure 7.

**Figure 8:**
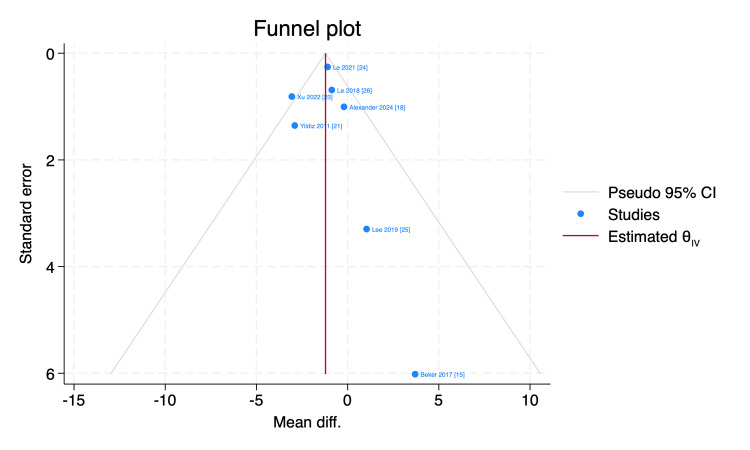
Funnel plot of the time needed to reach full sucking feeds with 95% CI. CI, confidence interval; Diff., difference

**Figure 9:**
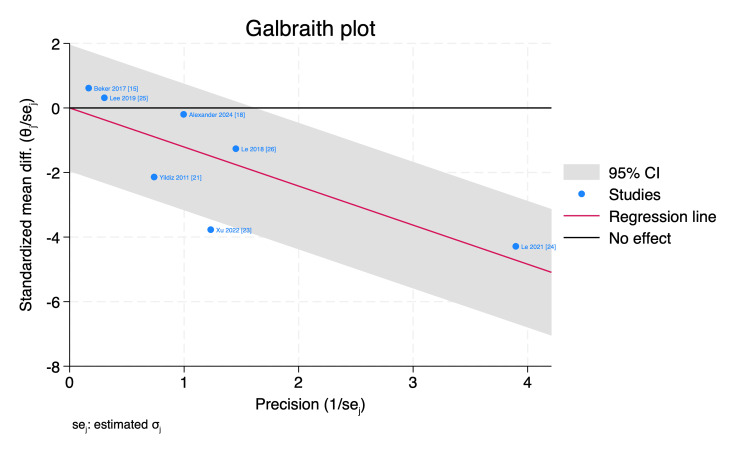
Galbraith plot of the time needed to reach full sucking feeds with 95% CI. CI, confidence interval; Diff., difference; SE, standard error

Funnel plot and Galbraith plot were used to assess the publication bias and the statistical heterogeneity, respectively, and by inspection, Xu et al. [23] visualized outside the 95% of the precision area, indicating its heterogeneity from other included studies, as shown in Figures 8, 9.

**Figure 10:**
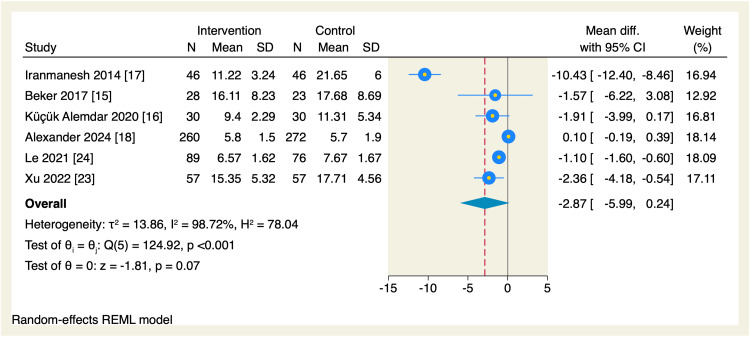
Forest plot of the time needed to reach enteral feeds. CI, confidence interval; Diff., difference; N, sample size; REML, restricted maximum likelihood; SD, standard deviation

**Figure 11:**
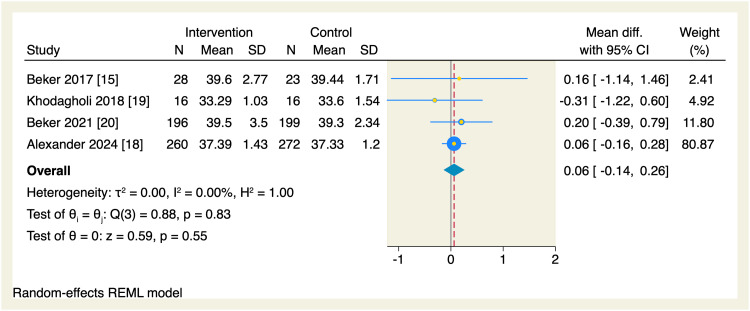
Forest plot of the postmenstrual age. CI, confidence interval; Diff., difference; N, sample size; REML, restricted maximum likelihood; SD, standard deviation

**Figure 12:**
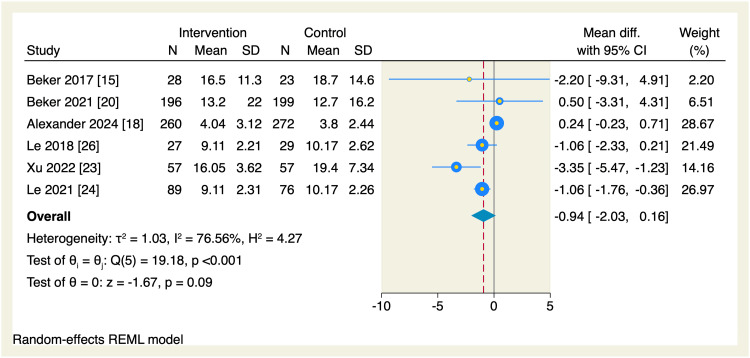
Forest plot of the need for parenteral nutrition. CI, confidence interval; Diff., difference; N, sample size; REML, restricted maximum likelihood; SD, standard deviation

**Figure 13:**
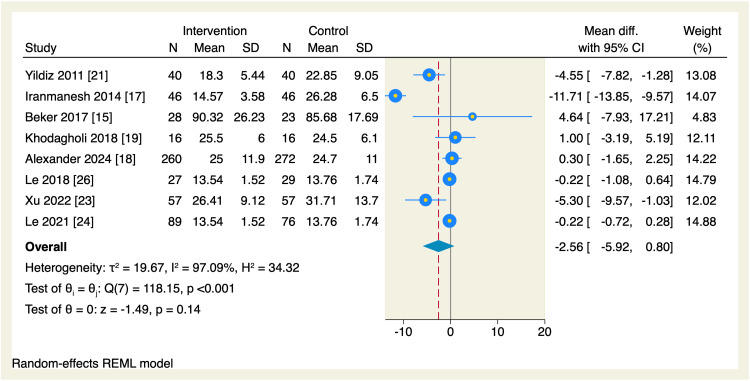
Forest plot of the length of hospital stay. CI, confidence interval; Diff., difference; N, sample size; REML, restricted maximum likelihood; SD, standard deviation

**Figure 14:**
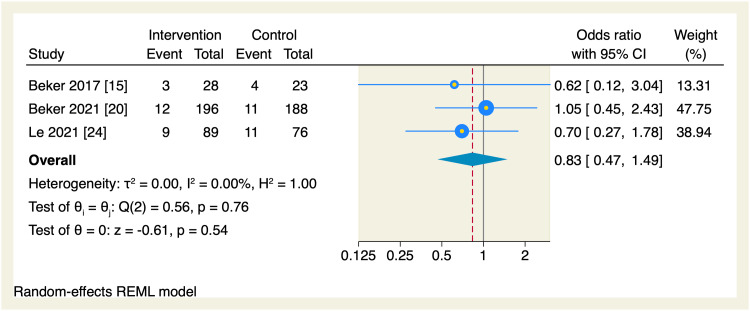
Forest plot of the incidence of necrotizing enterocolitis. CI, confidence interval; REML, restricted maximum likelihood

Regarding secondary endpoints, the pooled effect estimate showed no significant differences between the intervention and the control groups in terms of the time needed to reach enteral feeds (MD = -2.87 days, 95% CI [-5.99, 0.24], p = 0.07; I^2^ = 98.72, p < 0.001), change in postmenstrual age (MD = 0.06 weeks, 95% CI [-0.14, 0.26], p = 0.55; I^2^ = 0.00%), parenteral nutrition (MD = -0.94 days, 95% CI [-2.03, 0.16], p = 0.09; I^2^ = 76.56), length of hospital stay (MD = -2.56 days, 95% CI [-5.92, 0.8], p = 0.14; I^2^ = 97.09), and the incidence of necrotizing enterocolitis (OR = 0.83, 95% CI [0.47, 1.49], p = 0.54; I^2^ = 0.00%), as shown in Figures 10-14.

**Figure 15:**
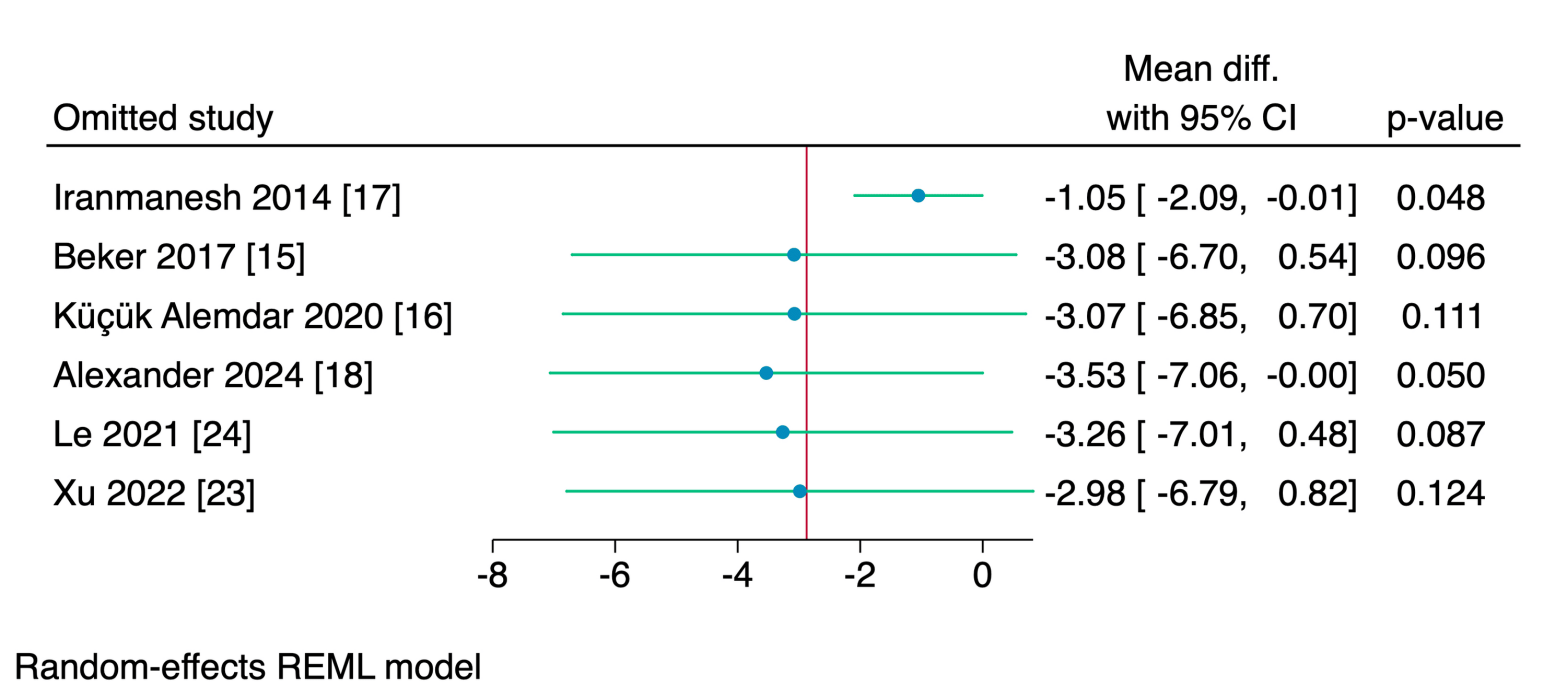
Leave-one-out sensitivity analysis plot of the time needed to reach enteral feeds with 95% CI. CI, confidence interval; Diff., difference

**Figure 16:**
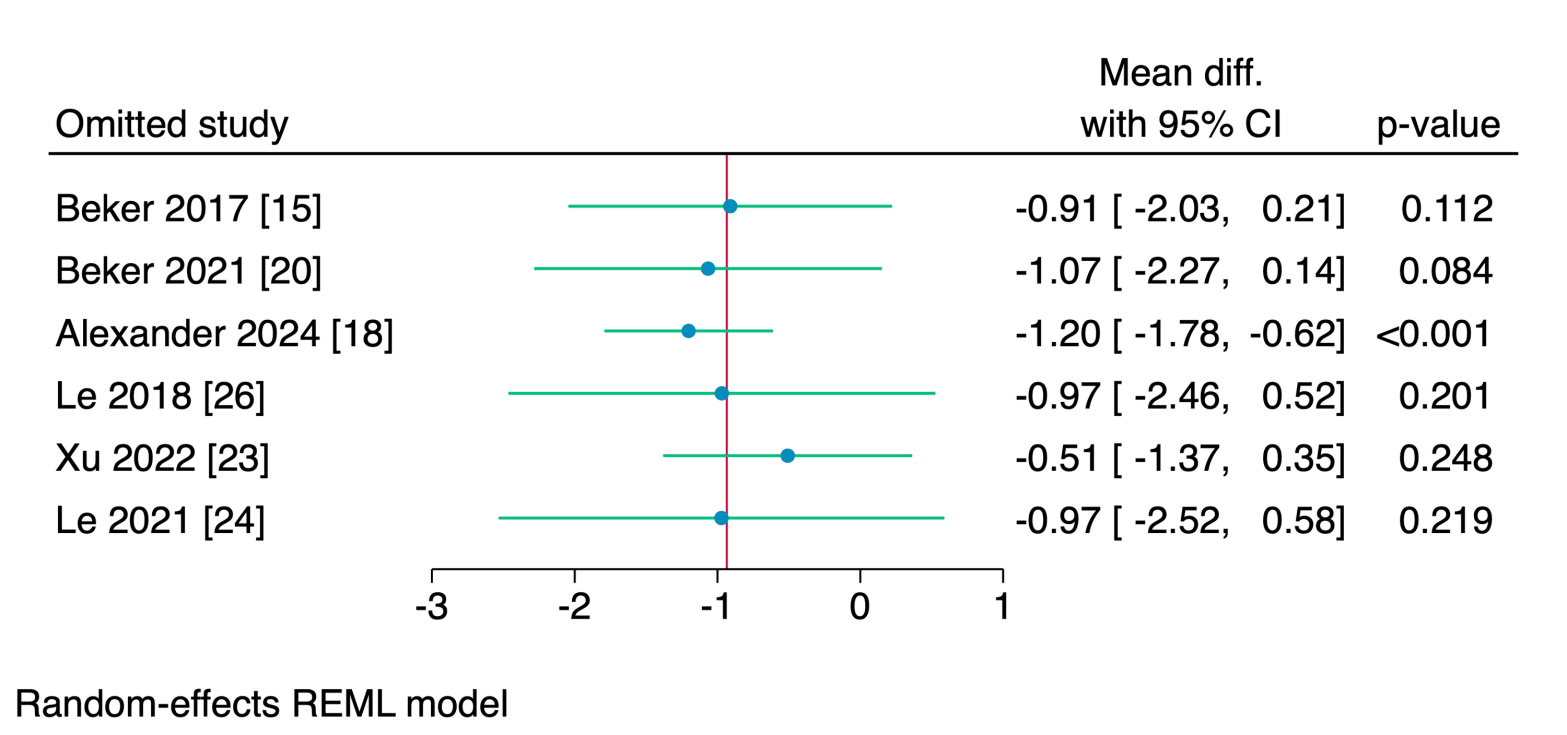
Leave-one-out sensitivity analysis plot of the need for parenteral nutrition with 95% CI. CI, confidence interval; Diff., difference

**Figure 17:**
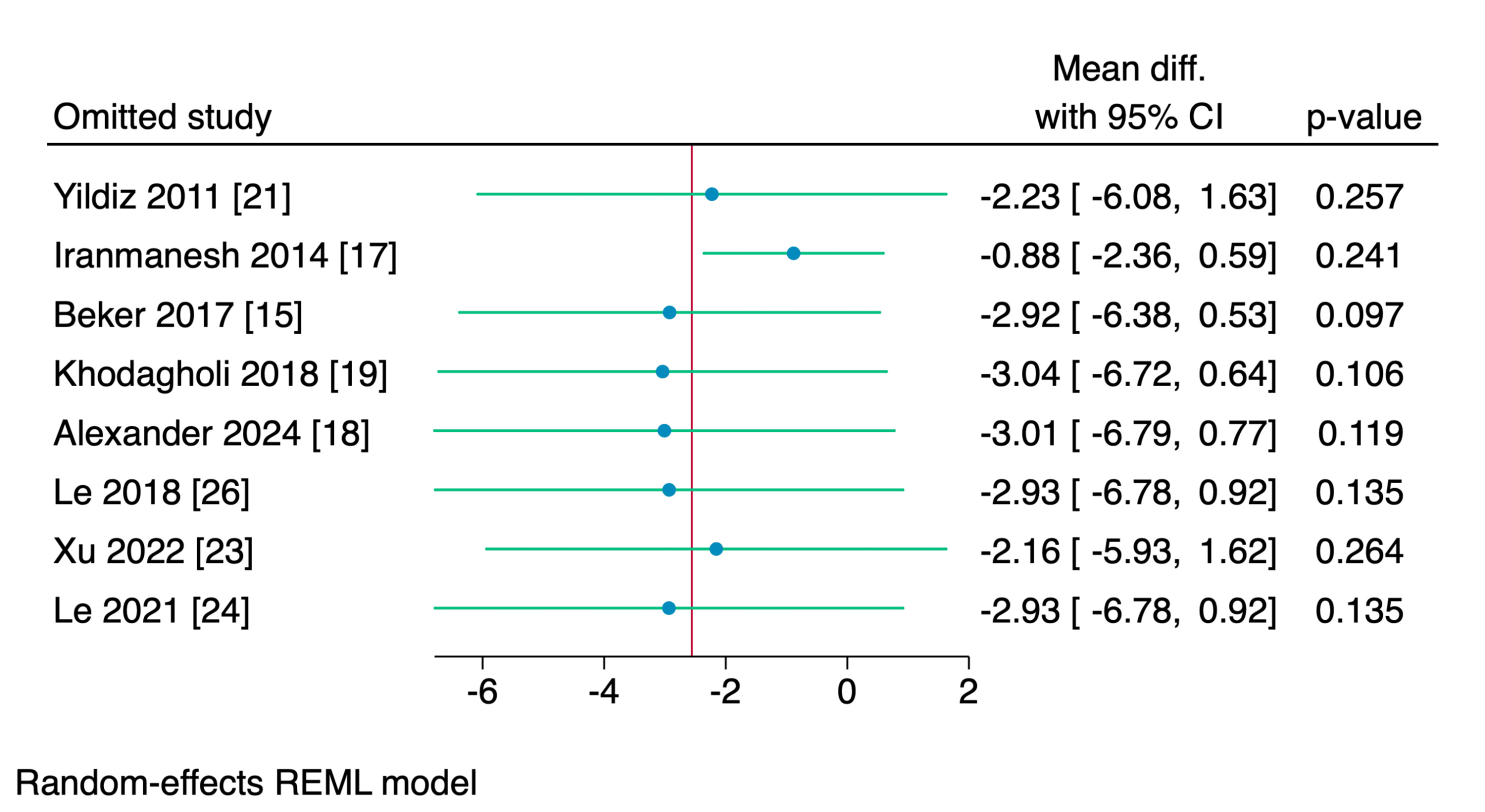
Leave-one-out sensitivity analysis plot of the length of hospital stay with 95% CI. CI, confidence interval; Diff., difference

Leave-one-out sensitivity analyses were performed for the time needed to reach enteral feeds, parenteral nutrition, and hospital stay, of which, upon excluding Iranmanesh et al. [17], the intervention group showed a significantly reduced amount of time needed to reach the enteral feedings (MD = -1.05 days, 95% CI [-2.09, -0.01], p = 0.048), and upon excluding Alexander et al. [18], the intervention group showed a significant reduction in the need of parenteral nutrition (MD = -1.2 days, 95% CI [-1.78, -0.62], p < 0.001); however, no single study had a disproportional effect on the overall effect estimate of the length of the hospital stay, as shown in Figures 15-17.

Discussion 

Our meta-analysis is the most comprehensive study to assess the impact of smell and taste sensation on the clinical outcomes of preterm infants aged 24-34 weeks of gestation including 12 RCTs comprising 1,638 infants. We found that smell and taste as an intervention was associated with a 1.37 day less time needed to reach the full sucking feeds compared to the control group. Moreover, our findings were consistent in cohorts with <1,500 g weight at admission, studies with >100 participants, and those allocated to the smell sensation only. However, we found no difference between the two studied groups regarding enteral feeds, postmenstrual age, parenteral nutrition, hospital stay, or the incidence of necrotizing enterocolitis. Additionally, sensitivity analyses showed that the intervention group was associated with a reduced amount of time needed to reach the enteral feedings and the need for parenteral nutrition.

Preterm infants tend to have longer NICU stays due to the severity of their medical condition, low gestational age, and low birth weight. Moreover, longer NICU stays were associated with severe complications as developmental delay, necrotizing enterocolitis, long-term separation of parent-infant relationship, and high medical costs related to the NICU stay [27] Therefore, a medical non-invasive intervention aiming to achieve faster maturation of oral feeding, which is the major determinant of the length of NICU stay, is warranted in such medical conditions [28]. Sensory stimulation and specific interventions such as the use of mother’s milk offered the benefit of allowing mothers to take care of their preterm infants [28].

In our study, we found that the smell and taste of mother’s milk reduced the time needed to reach oral feeds compared to the no-intervention group. In alignment with our findings, Bingham et al. [29] found that smell stimulation via mother’s milk increased the nutritive sucking during gavage feeding. Although the sensation of chemosensory stimulators was unclear, the stimulation of retronasal olfactory cells during the gavage feeding might have affected the behavior of gavage feeding positively [29].

Subgroup analysis based on the type of exposure in our study revealed that only the smell subgroup had better outcomes compared to the combination of smell and taste intervention to reach the oral feeds. In another study by Yildiz et al. [21], the smell intervention was associated with a lower time needed to reach oral feeds in the intervention arm, highlighting that the success to reach oral feeds depends on the successful coordination of four mechanisms: sucking, respiration, swallowing, and the development of neurological maturation of muscles responsible for sucking techniques [21]. Moreover, stimulation of smell of odor via mother’s milk led to an earlier maturation of preterm infants and the sucking behavior. Additionally, although preterm infants tend to have lower birth weight, introducing nutritive odors could graduate preterm infants to earlier oral feeding [30]. This finding was proved by our subgroup analysis based on the birth weight at admission, of which preterm infants who had a birth weight of <1,500 g tend to benefit much more and to reach the full sucking feeds compared to those who had a birth weight of >1,500 g at admission.

We found that the time needed to reach enteral feeds was comparable between the intervention and no-intervention groups, although upon sensitivity analysis by excluding Iranmanesh et al. [17], the intervention group showed a decrease in the time needed to reach enteral feeds. The current finding was aligned with previous studies that demonstrated similar effects on the odor or taste interventions compared to the control group [31]. In a study by Beker et al. [20], there was no significant difference between the two groups regarding the time to reach enteral feeds; however, in an adjusted survival analysis, there was a trend of benefit in preterm infants who were allocated to regular smell and taste intervention. Their findings could be attributed to the enhanced cephalic-phase response, improvement in absorption, and metabolism of nutrients and digestion, all of which aided in the regulation of appetite in their preterm infants.

Although the mechanism of cephalic-phase response is yet to be examined, some indications such as brain tissue oxygenation tend to change in circumstances related to the exposure to pleasant or unpleasant smells [32,33]. Moreover, the current finding confirms the greater tendency of the preterm infants to breastfeed once they were fed from their mother’s milk for the first time, which could be attributed to the improvement in sucking skills via several olfactory stimulation sessions and the independent behavior of the preterm infant to feed after the end of the stimuli, suggesting the ability of the infants to continue oral feeding at a lower PMA [34].

Our results are in alignment with previous systematic reviews and meta-analyses suggesting that smell and taste sensation might aid in reducing the time needed to reach oral and enteral feeds [31,35]. On the other hand, a systematic review and meta-analysis by Cochrane [36] found little to no effect on the time needed to reach oral or enteral feeds with very low-certainty evidence. The difference from our reported results may be due to their inclusion of only seven RCTs including 1,244 infants and we included 12 RCTs comprising 1,747 infants. Additionally, we tested the primary outcome to subgroup analyses based on the birth weight at admission, and the sample size of each included RCT that were not studied in the previous meta-analyses.

Our study highlighted the exposure of smell and taste sensation to preterm infants during the NICU stay owing to their beneficial effects on the time needed to reach oral feeds and the time needed to reach enteral feeds. Although we found no significant difference in terms of hospital stay, significant heterogeneity was found, which could alleviate the results. Also, we found no difference regarding the incidence of necrotizing enterocolitis, which is the major determinant of NICU mortality in preterm infants. We, therefore, highlight the beneficial effect of smell and taste sensation in preterm infants, and clinicians should pay high attention to smell and taste experience, considering the necessity of food, which is vital for human beings.

This research has several limitations, including the small number of studies included and the small corresponding sample size. Additional limitations involve the lack of adequate information in many studies regarding key baseline characteristics, such as head circumference and APGAR scores at 1 and 5 minutes; however, we performed key sensitivity analyses on birth weight, type of exposure, and sample size to account for the heterogeneity found. Furthermore, nearly half of the studies raised "some concerns of bias," which could lower the impact of the current findings.

## Conclusions

In the meta-analysis including 12 RCTs, we observed that smell and taste sensation was associated with a reduced time needed to reach full sucking feeds, with a safe profile concerning necrotizing enterocolitis. These findings suggest that sensory interventions could be a valuable addition to the care of preterm infants. However, given the heterogeneity in study designs and sample sizes, large-volume RCTs with more robust methodologies and long-term follow-up are warranted to confirm and further validate the current findings. Such studies should also explore the potential long-term developmental impacts of these interventions on preterm infants.
